# Supplementary material for: PDX models recapitulate the genetic and epigenetic landscape of pediatric T‐cell leukemia
Source: EMBO Mol Med. 2018 Nov 2;10(12):e9443. doi: 10.15252/emmm.201809443 (PMC6284381; doi:10.15252/emmm.201809443)
Supplement: Supplementary file 1 — Appendix [file EMMM-10-e9443-s001.pdf]

APPENDIX

Table of Content:

1. **Appendix Figure S1**

   SNP fingerprinting heatmap of original patients' samples and their corresponding PDX models shows identical SNP patterns. Four samples derived of each patient cluster together.

2. **Appendix Figure S2**

   Sanger sequencing chromatograms of gene fusion regions in genomic DNA of primary patients' material for P1: fusion *NKX2-4*:*TRBC2*; P9: fusion *TLX1:TRBC2* and P11: fusion *LMO2:TRDJ*.

3. **Appendix Figure S3**

   Total number of SNVs/InDels (AF≥10%) detected in primary samples (x-axis) plotted against the number of SNVs/InDels detected in corresponding PDX samples (y-axis).

4. **Appendix Figure S4**

   Coverage segmentation plots of copy number alterations detected in primary samples and corresponding segmentation plots of chromosomes of matched PDX models generated by low coverage whole genome sequencing (2-3 reads/bp).

5. **Appendix Figure S5**

   Sanger sequencing chromatograms showing validation of five mutations (a: N642H in *STAT5B*, R211Q in *FOXO3*, R199Q in *DNM2*, R1023Q in *PIK3CA* and T908K in *MAP1B*) detected by whole exome sequencing in the primary patients' samples, but not in the corresponding PDX samples; validation of six mutations (b: L1585P in *NOTCH1*, R137H in *SGCE*, V374M in *COL6A3*, V222I in *WDR88*, 1617+17del in *MAST3* and E665G in *PCDH15*) detected in five PDX samples, but not in the matched primary patients' samples.

6. **Appendix Figure S6**

   Composite plot of regional methylation of the promoters in primary patients' samples and in corresponding PDX models. Relative coordinates of 0 and 1 corresponds to the start and end coordinates of the promoter. Coordinates smaller than 0 and greater than 1 denote flanking regions normalized by region length. Horizontal lines indicate region boundaries. For smoothing, generalized additive models with cubic spine smoothing were used. Deviation bands indicate 95% confidence intervals.

7. **Appendix Figure S7**

   Representative ATAC-seq tracks (all tracks within one track panel were adjusted to the same value to facilitate direct visual comparison).

   A Examples (*TLX3*, *NOTCH1* and *IL7R*) of known leukemia drivers that are accessible in both, primary and PDX samples.

   B Examples (*MAG1*, *SLC22A23* and *SUSD4*) of regions significantly more accessible in PDX samples.

C, D Examples (*GATAD2B*, *ZNF250* and *MAPK14*) of regions significantly more accessible in primary samples (**C**) related to the category of cytokine signaling **(D)**.

8. Appendix Figure S8

Allele frequencies (AF) of the mutations detected in relapse samples (x-axis) and in PDX models derived of the matched samples collected at initial diagnosis (y-axis).

Appendix Figure S1

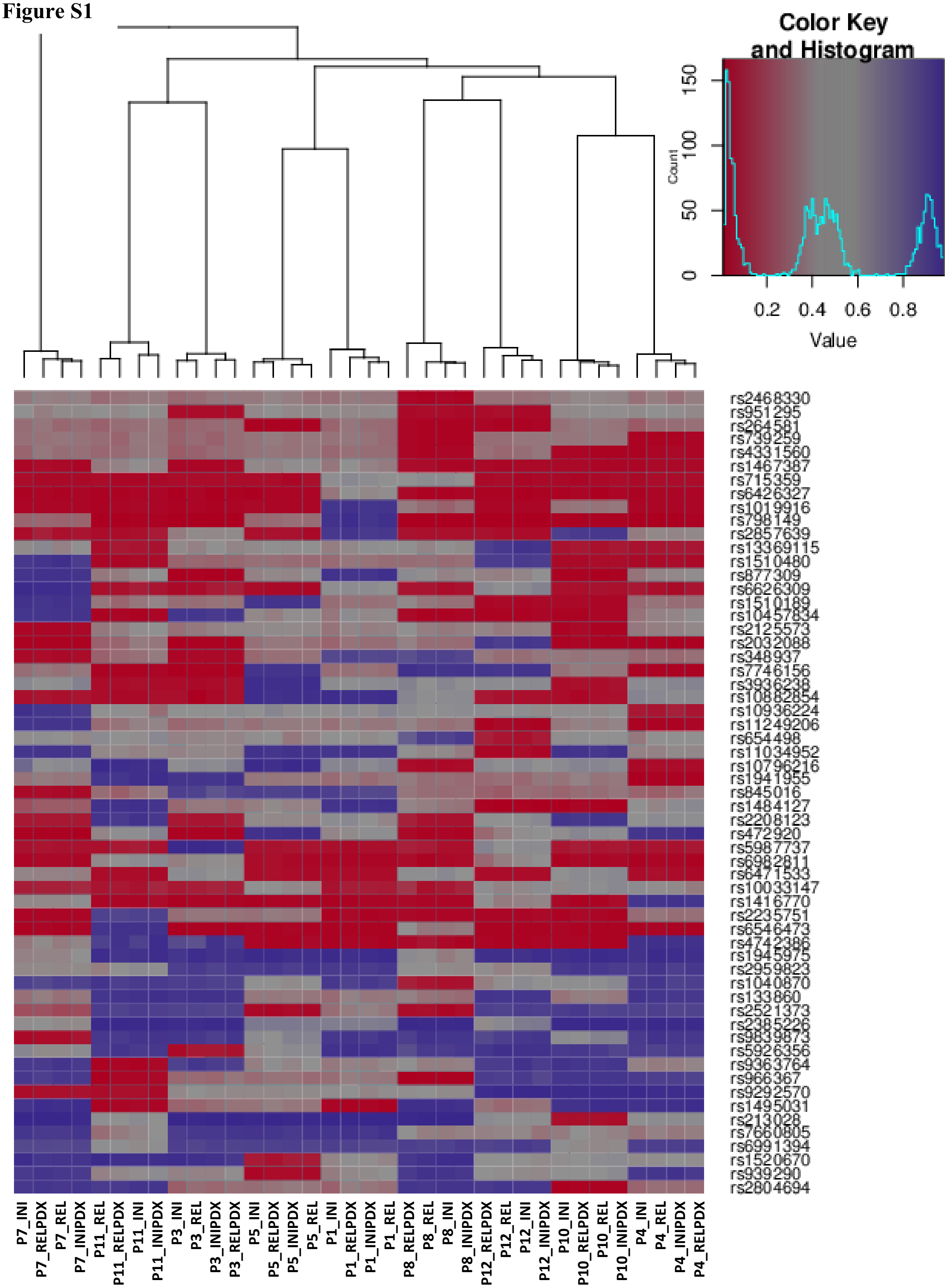

Appendix Figure S2

P1: fusion NKX2-4:TRBC2

NKX2-4 random nts **TRBC2**

TGACAGCGAGGGCGCCCCGGGGCTAGGTCCTGGTG | TGG | GCAGTACTTCGGGCCGGGCACCAGGCTCACGGT

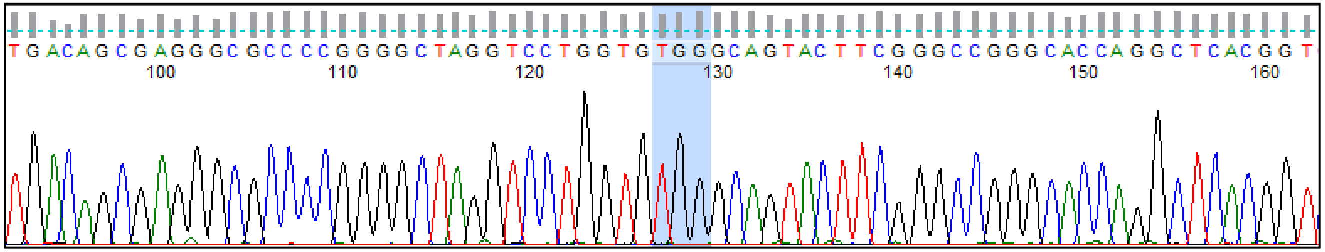

P9: fusion TLX1:TRBC2

TLX1 random nts **TRBC2**

CTCCCTCCAGCACAAACACAAGGTCATGGCC | TAAGGATGTT | CTCCTACAATGAGCAGTTCTTCGGGCCAGGG

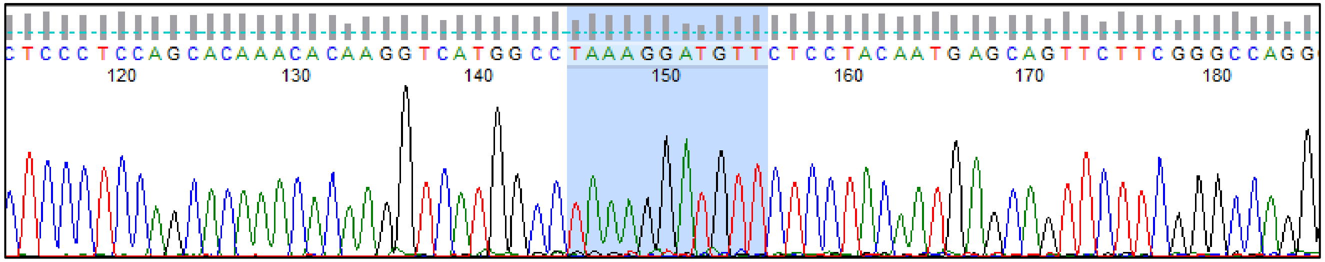

P11: fusion LMO2:TRDJ3

LMO2 random nts **TRDJ3**

tattcataggctgtgacttggagaggcccc | GATGTAGAT | TCCTGGGACACCCGACAGATGTTTTTCGGAA

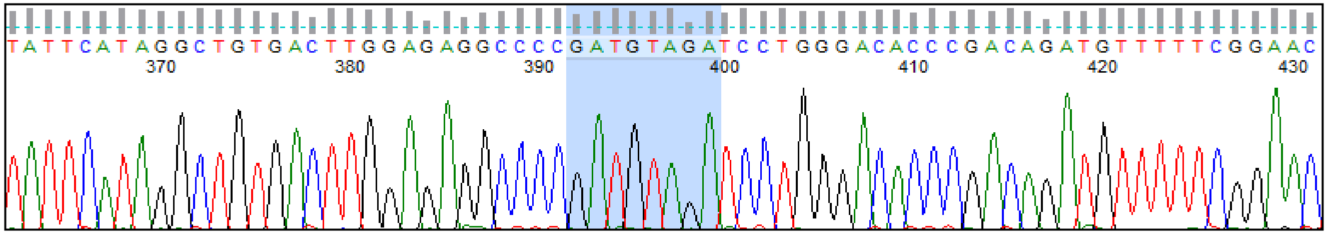

Appendix Figure S3

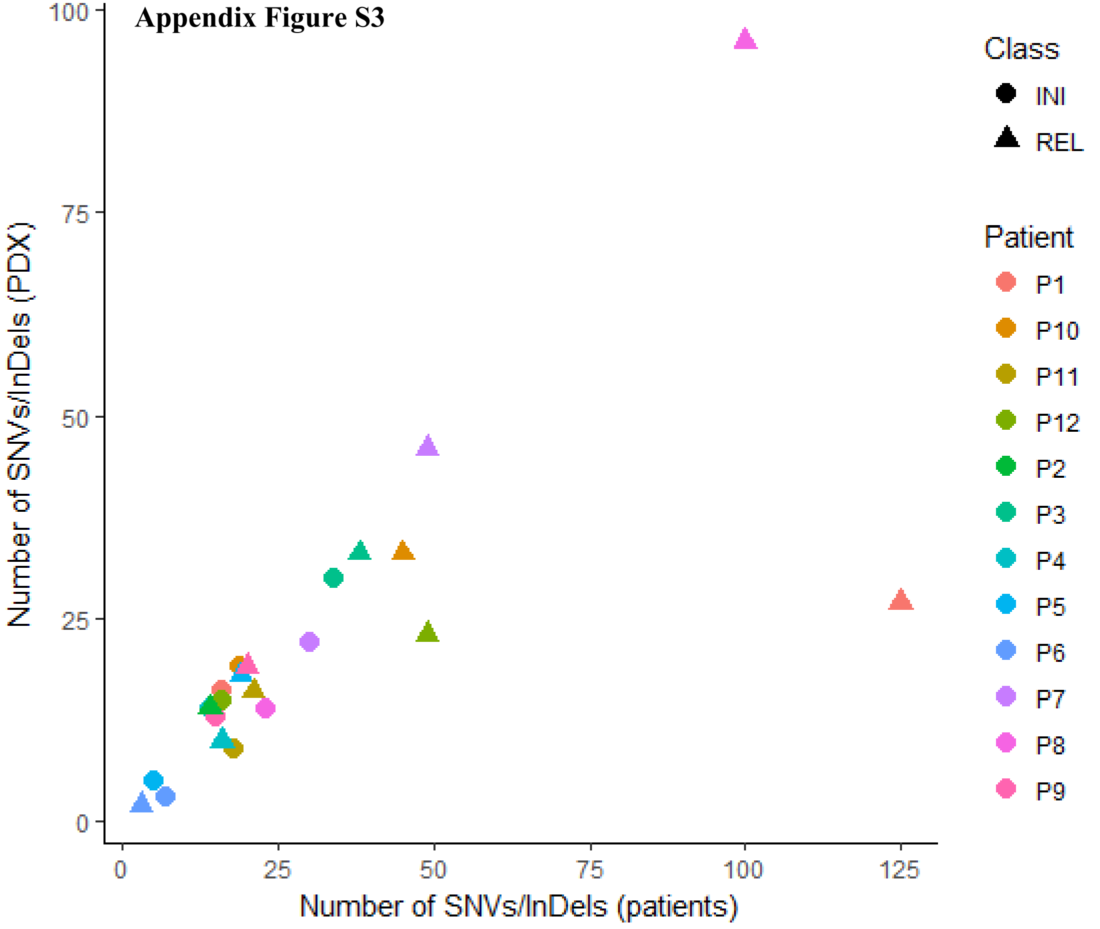

Appendix Figure S4

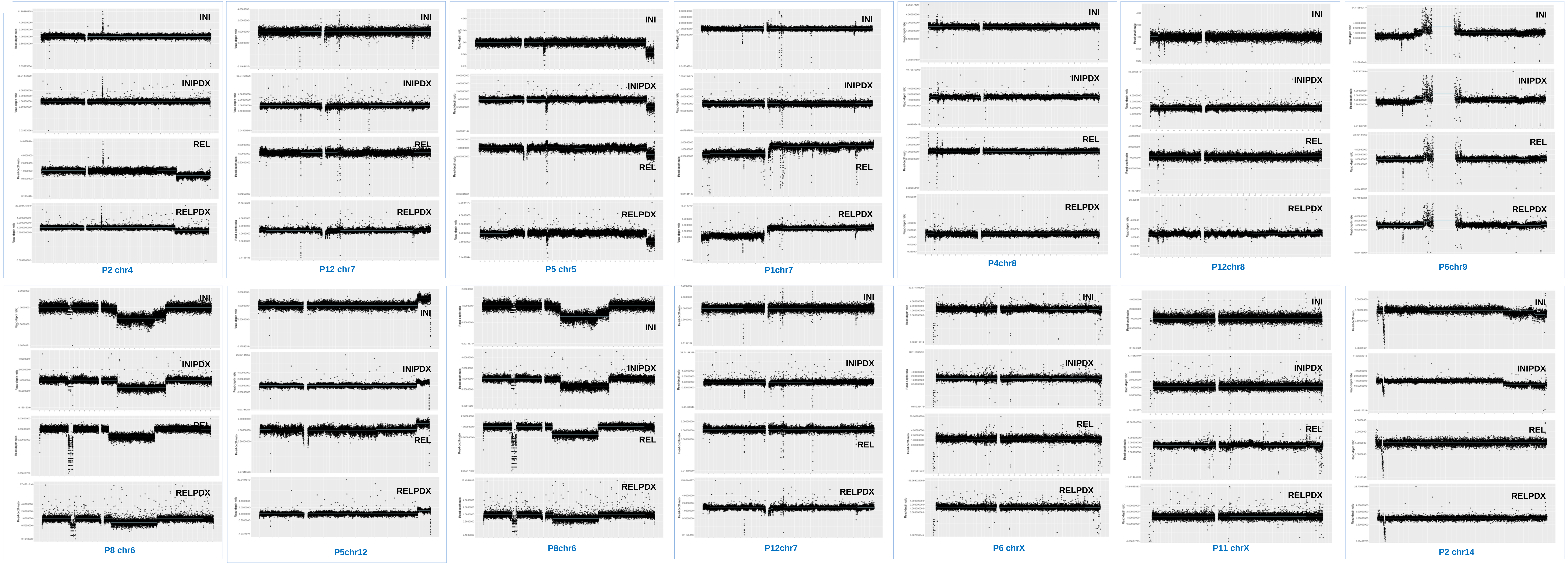

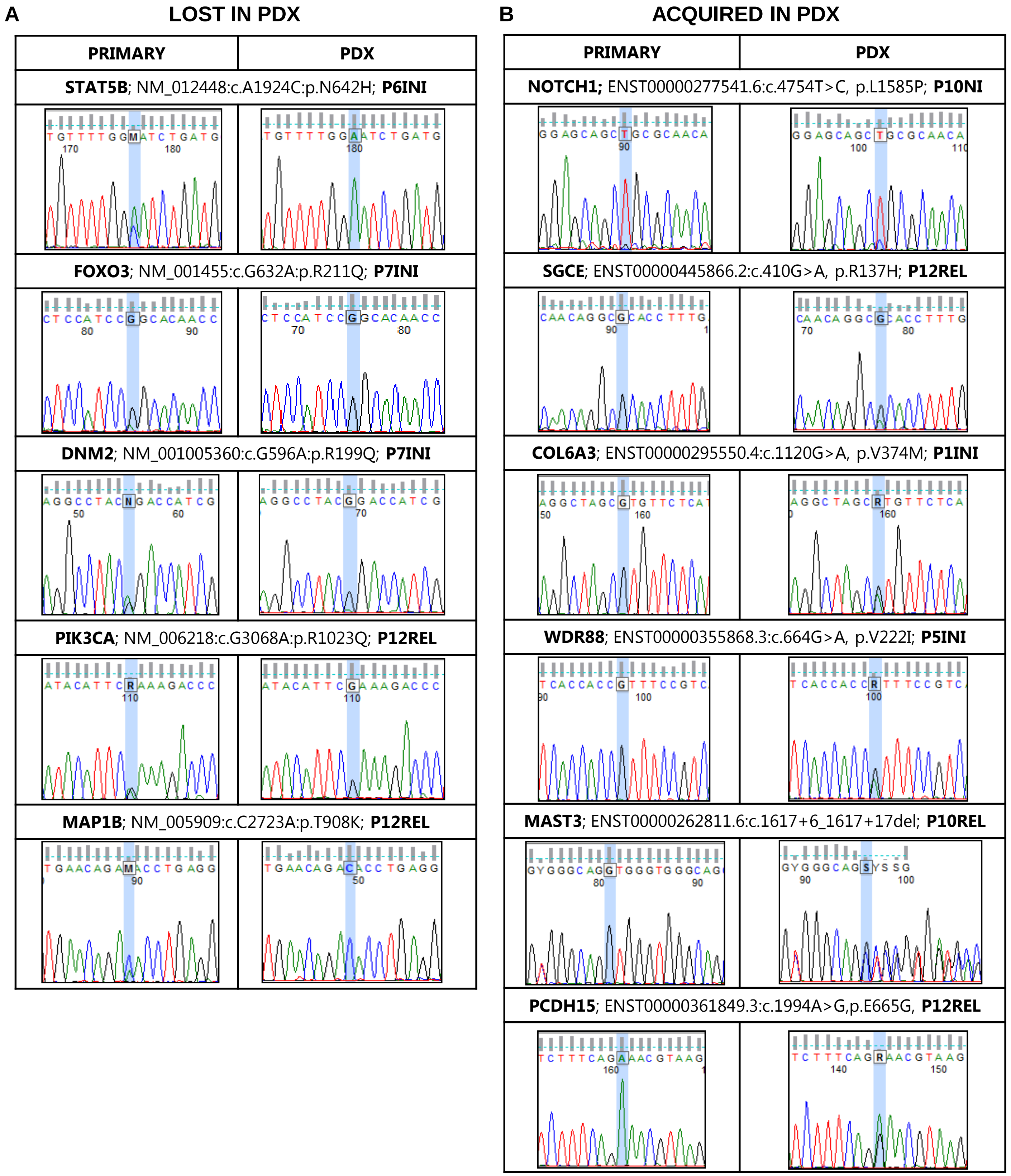

Appendix Figure S5

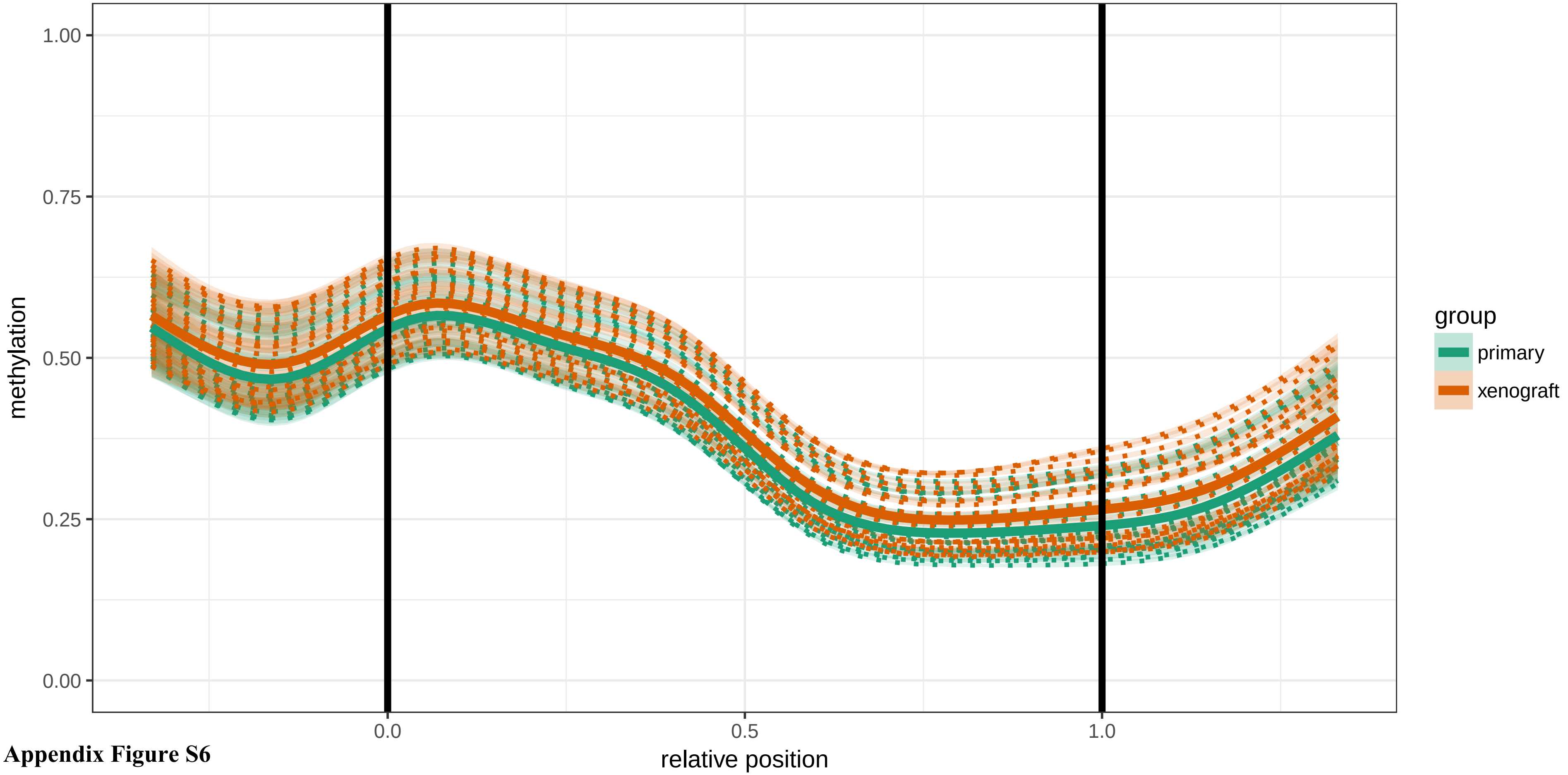

Appendix Figure S6

Appendix Figure S7

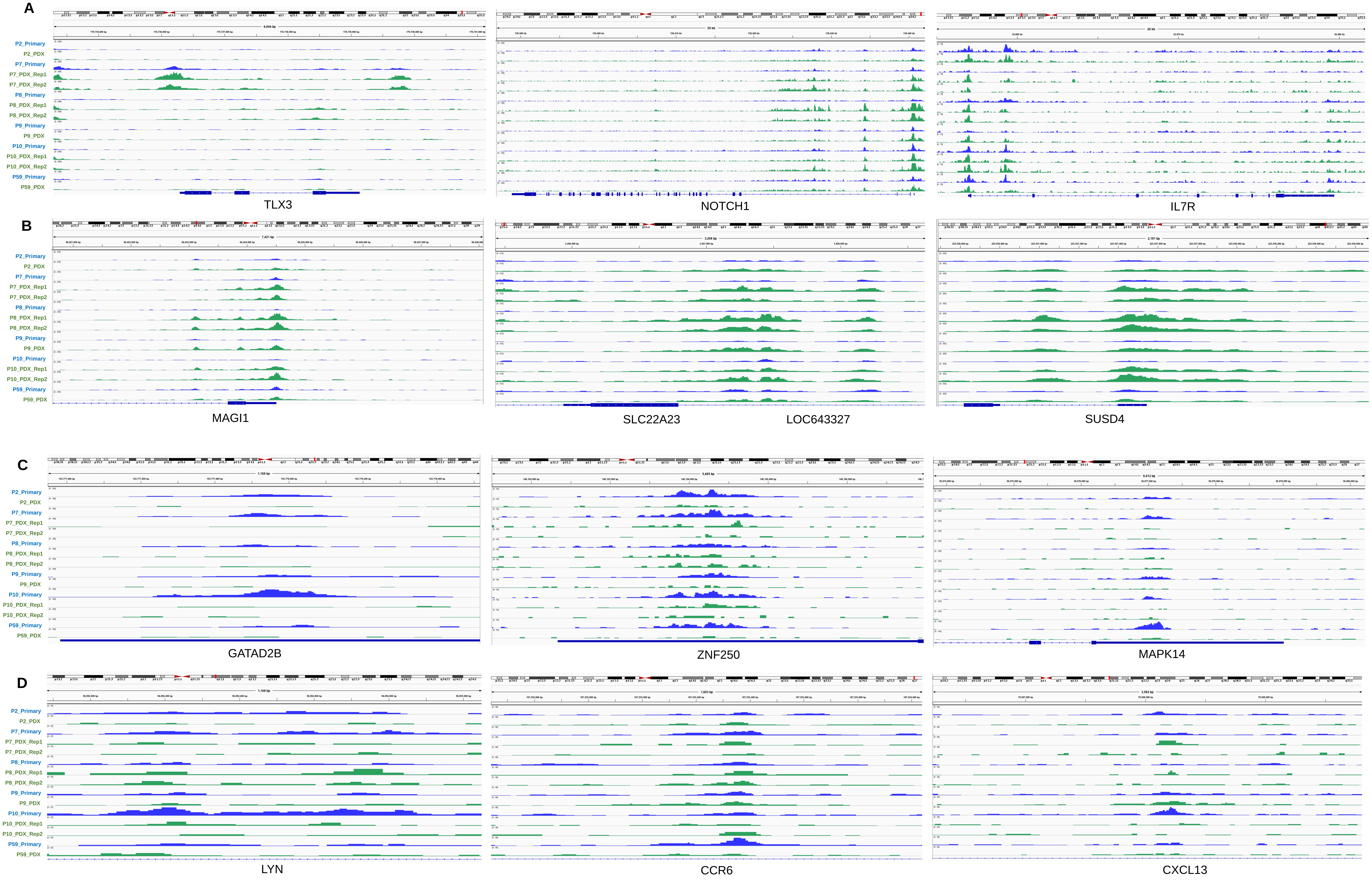

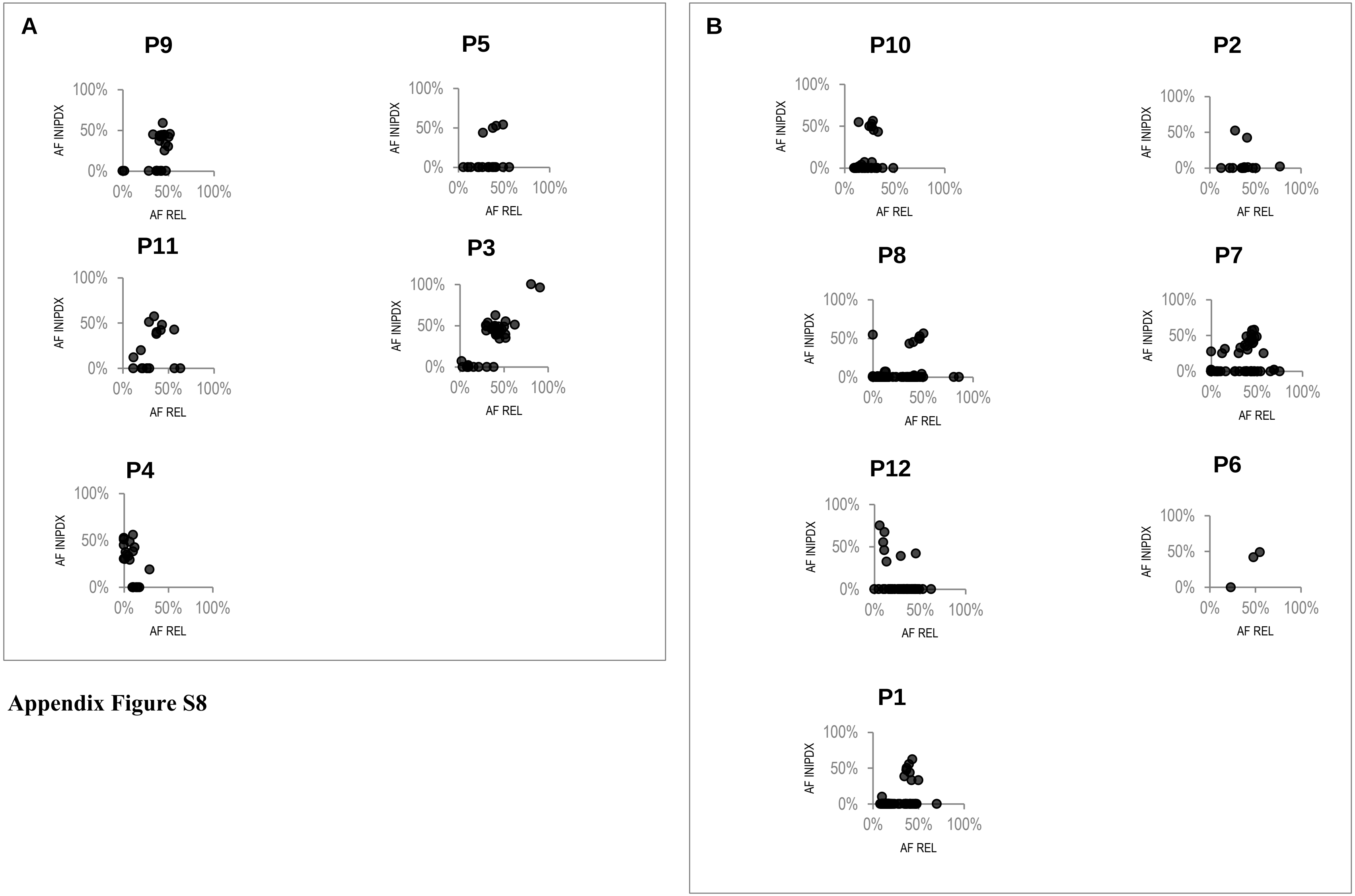

Appendix Figure S8
