# Supplementary material for: PDX models recapitulate the genetic and epigenetic landscape of pediatric T‐cell leukemia
Source: EMBO Mol Med. 2018 Nov 2;10(12):e9443. doi: 10.15252/emmm.201809443 (PMC6284381; doi:10.15252/emmm.201809443)
Supplement: Supplementary file 2 — Expanded View Figures PDF [file EMMM-10-e9443-s002.pdf]

Expanded View Figures

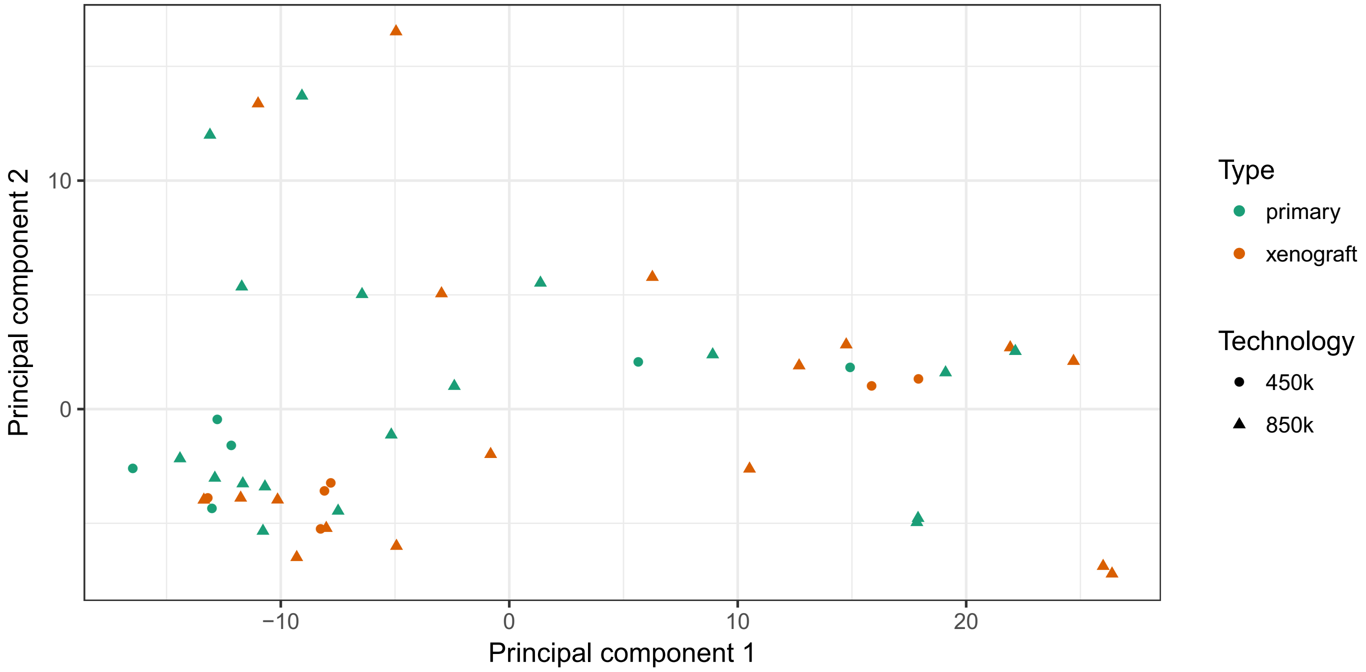

Figure EV1. PCA shows that the use of two different arrays (450k and 850k) did not introduce any bias.

Scatter plot showing the samples' coordinates on principal components 1 and 2. Principal component analysis based on the average methylation of the promoters in the performance of the 450k array (circles) and the 850k (EPIC) array (triangles).

 © 2018 The Authors

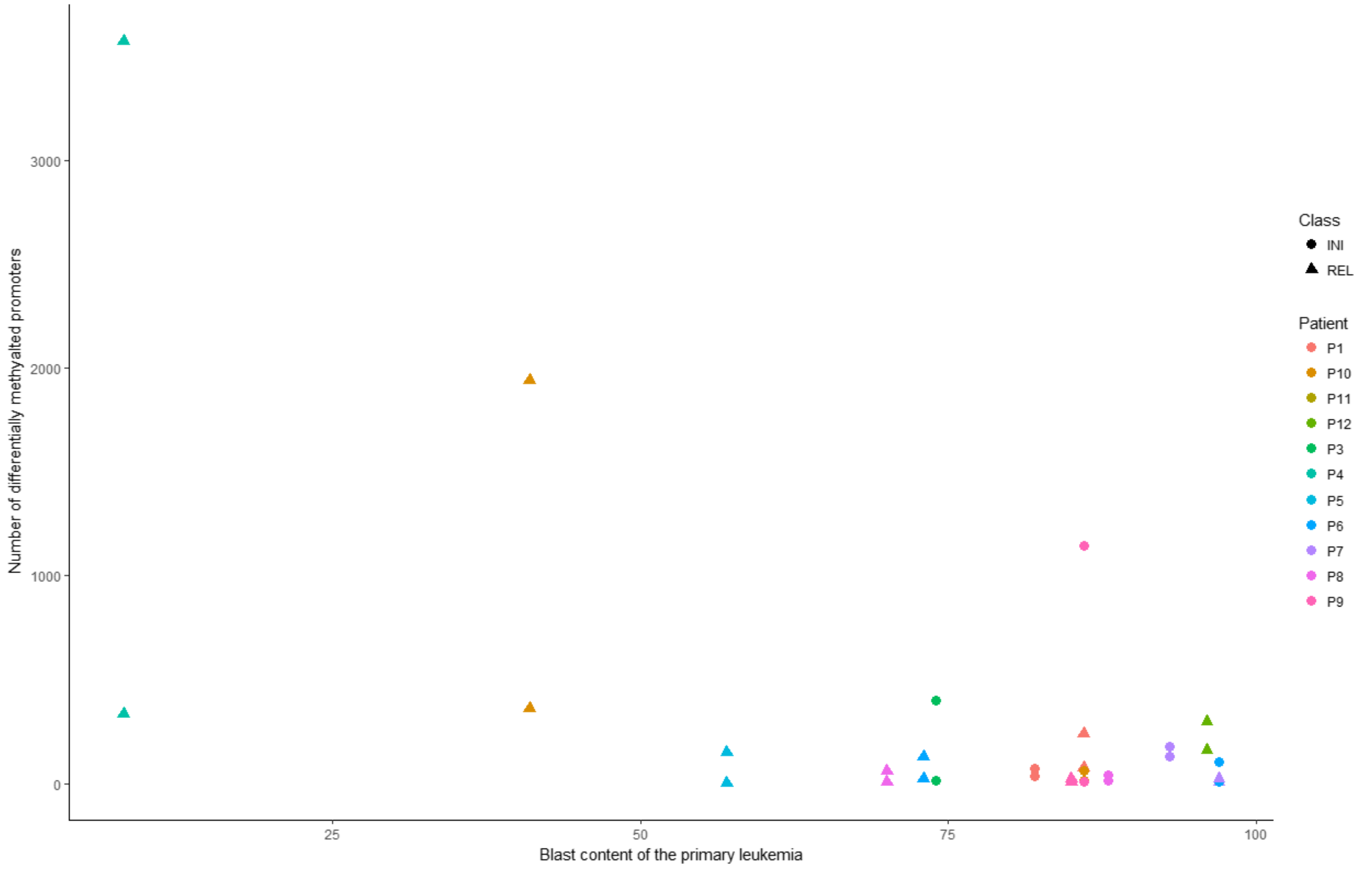

Figure EV2. Negative correlation between the blast content and the total number of differentially methylated promoters.

Total number of changes in methylation levels (min. difference in β value = 0.2) between primary samples (PRI) and the PDX models per sample plotted against the blast content of the sample.

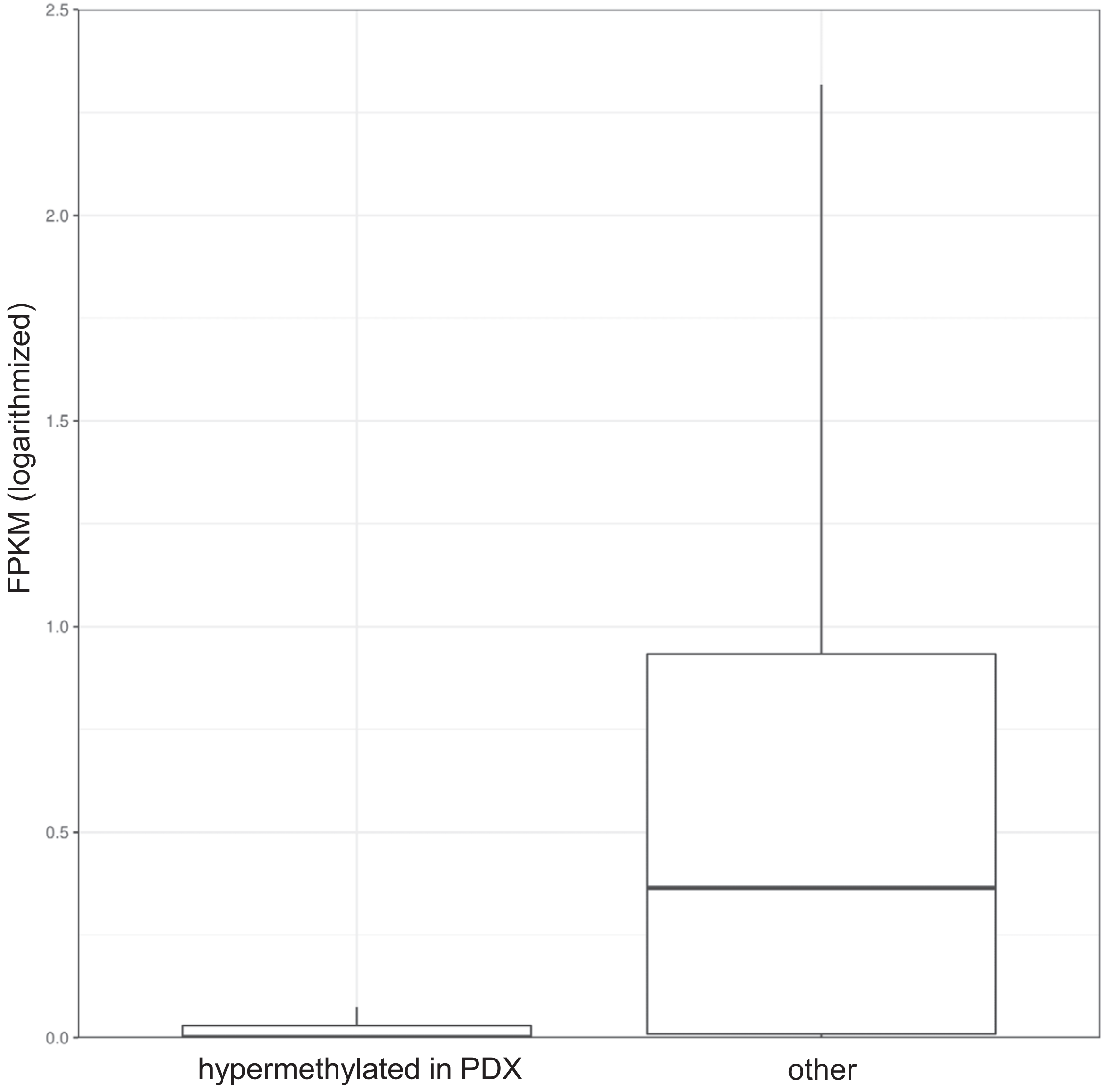

Figure EV3. Majority of hypermethylated genes are transcriptionally silent in T-ALL.

Boxplot comparing expression values (FPKM) between the genes that were hypermethylated upon propagation in mice (22) and the entire transcriptome in the cohort of 264 publicly available primary patients' samples (Liu *et al*, 2017). Horizontal lines indicate median, lower and upper limits of each box correspond to the first and third quartiles (the 25th and 75th percentiles) and the lower and upper whiskers extend from min to max..

 © 2018 The Authors

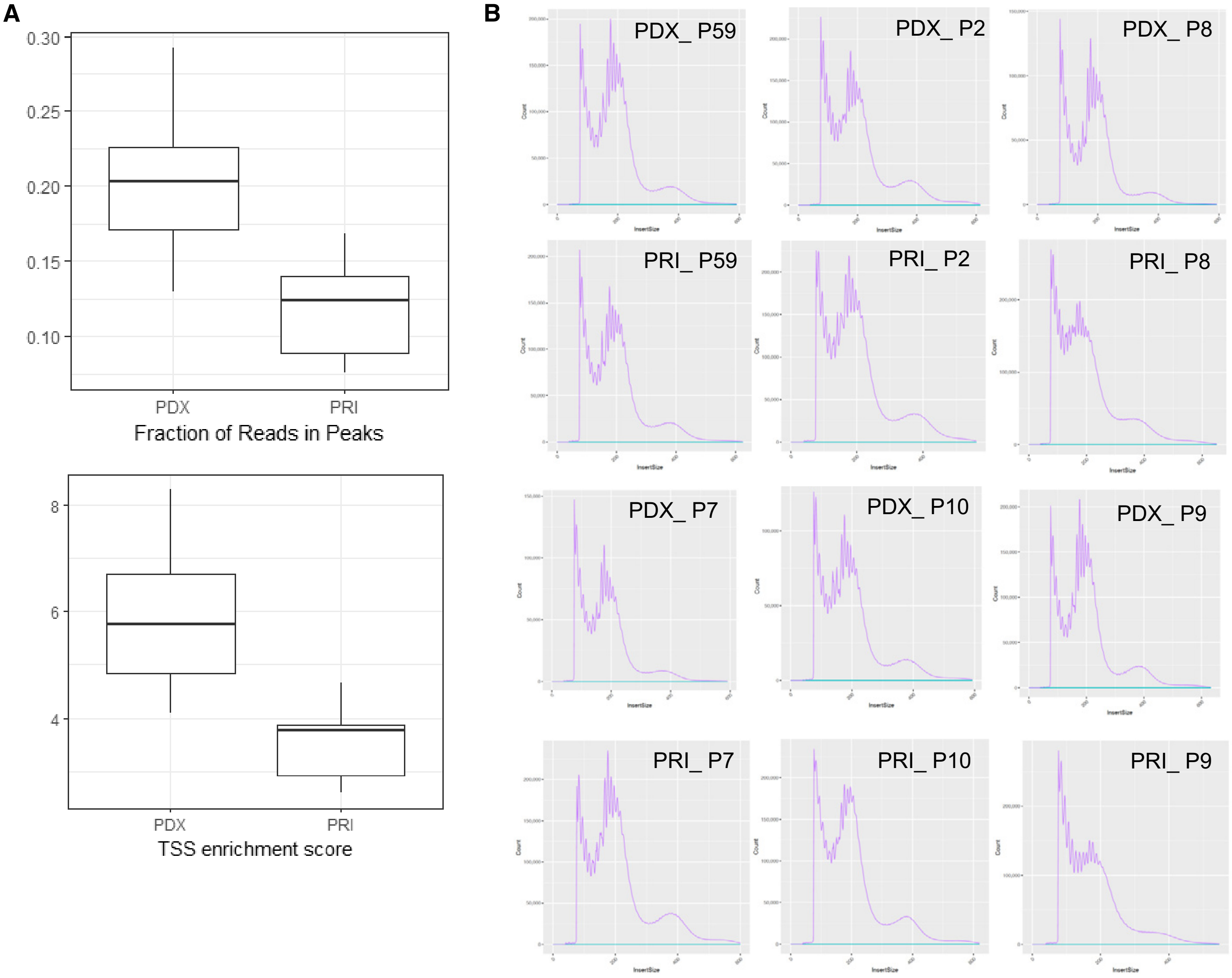

Figure EV4. ATAC library quality control metrics.

A, B Comparison of (A) fraction of reads in peaks, transcription site enrichment score, and (B) insert size distributions between the six matched pairs of the primary samples (PRI) and of PDXs. Details of the QC analysis can be found at https://github.com/tobiasrausch/ATACseq. Horizontal lines of the boxplots indicate median, lower and upper limits of each box correspond to the first and third quartiles (the 25th and 75th percentiles) and the lower and upper whiskers extend from min to max.

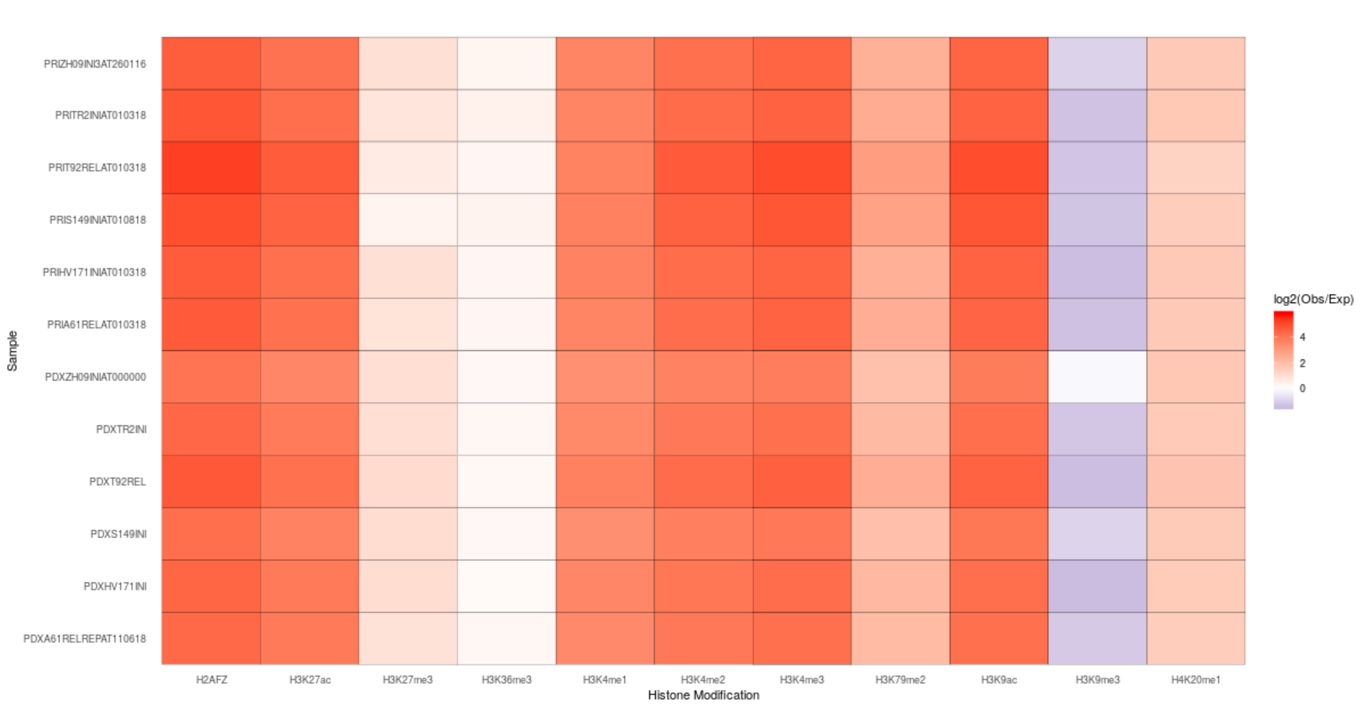

Figure EV5. Comparison of the ATAC peaks with the chromatin immuno-precipitation DNA-sequencing.

Heatmap showing degree of overlap between the ATAC peaks and the active promoters and enhancers detected in histone methylation/acetylation analysis by chromatin immunoprecipitation and sequencing of the cell line DND-41. Expected values were computed based on the randomly shuffled peaks.

 © 2018 The Authors
